# Astrocyte Atrophy and Immune Dysfunction in Self-Harming Macaques

**DOI:** 10.1371/journal.pone.0069980

**Published:** 2013-07-26

**Authors:** Kim M. Lee, Kevin B. Chiu, Hope A. Sansing, Fiona M. Inglis, Kate C. Baker, Andrew G. MacLean

**Affiliations:** 1 Tulane National Primate Research Center, Covington, Louisiana, United States of America; 2 Tulane Program in Biomedical Science, Tulane University School of Medicine, New Orleans, Louisiana, United States of America; 3 Department of Biomedical Engineering, Tulane University, New Orleans, Louisiana, United States of America; 4 Department of Psychology, Tulane University, New Orleans, Louisiana, United States of America; 5 Tulane Program in Neuroscience, Tulane University, New Orleans, Louisiana, United States of America; 6 Department of Cell and Molecular Biology, Tulane University, New Orleans, Louisiana, United States of America; 7 Department of Microbiology and Immunology, Tulane University School of Medicine, New Orleans, Louisiana, United States of America; University of Jaén, Spain

## Abstract

**Background:**

Self-injurious behavior (SIB) is a complex condition that exhibits a spectrum of abnormal neuropsychological and locomotor behaviors. Mechanisms for neuropathogenesis could include irregular immune activation, host soluble factors, and astrocyte dysfunction.

**Methods:**

We examined the role of astrocytes as modulators of immune function in macaques with SIB. We measured changes in astrocyte morphology and function. Paraffin sections of frontal cortices from rhesus macaques identified with SIB were stained for glial fibrillary acidic protein (GFAP) and Toll-like receptor 2 (TLR2). Morphologic features of astrocytes were determined using computer-assisted camera lucida.

**Results:**

There was atrophy of white matter astrocyte cell bodies, decreased arbor length in both white and gray matter astrocytes, and decreased bifurcations and tips on astrocytes in animals with SIB. This was combined with a five-fold increase in the proportion of astrocytes immunopositive for TLR2.

**Conclusions:**

These results provide direct evidence that SIB induces immune activation of astrocytes concomitant with quantifiably different morphology.

## Introduction

Worldwide, suicide attempts, plans, and ideation are found in approximately 3% of the population annually [1]. In the United States, suicide is the eighth leading cause of death among adults, which rises to third among adolescents [2]. Suicide is closely associated with both anxiety and depressive disorders, as well as the comorbid condition [3], [4]. Self-harm, which is diagnosed in approximately 4% of the population not affected by neurodevelopmental disorders [5], [6], is significantly associated with suicide [7], [8]. We have recently used a nonhuman primate model for self-harm: self-injurious behavior (SIB) in rhesus macaques. Approximately 5–13% of the caged population spontaneously demonstrate SIB in the form of self-biting [9], [10]. While self-biting may appear severe to an observer, skin trauma is atypical. However, occasionally if a macaque engages in a more extreme form of SIB, veterinary intervention may be required [11]. These animals respond well to the antidepressants naltrexone and fluoxetine, further indicating the relevance of this behavior in the nonhuman primate model of aberrant behavior [11].

It is known that stress can affect the plasticity of glial cells including astrocytes [12]. Historically, astrocytes have been perceived as “nerve glue”, albeit cells with heterogeneous morphology depending on location [13]. Astrocytes comprise a heterogeneous population of cells with at least 9 different morphological variants that can coexist within a given brain region [13], [14]. Distinct immunohistochemical markers classify these variants, so for our purposes, we classified our astrocytes into two main categories: protoplasmic astrocytes, which are abundantly found in gray matter and characterized by highly complex processes, and fibrous white matter astrocytes, which have clear processes and moderate branching [15]. Recently, we have demonstrated the important relationship between astrocyte morphology and activation [16]. There are significant differences in arbor, cell body size and number of processes of frontal cortex white matter astrocytes during lentiviral infection (Lee (a), under review) and in the anterior cingulate in suicidal humans [17].

Indirect mechanisms for neuropathogenesis of aberrant behavior are being considered including irregular immune activation, host soluble factors, and astrocyte dysfunction. In addition to providing metabolic and trophic support, astrocytes are involved in the maintenance of the blood-brain barrier (BBB), neuronal homeostasis and transmission, and neuroprotection [18]. Astrocyte activation and gliosis ultimately lead to BBB dysfunction [19], [20], monocyte/macrophage infiltration [20], [21], and aberrant cytokine/chemokine production [22]. The cellular mechanisms by which astrocytes are activated in behavioral disorders, and how this conveys a signal to neurons and endothelial cells remain to be elucidated in detail.

There are two distinct mechanisms whereby astrocytes can be activated in the absence of infectious agents. In the first, gap junction proteins are down regulated [23] restricting the overall syncytia of astrocytes. This would also alter the morphology of the astrocytes including the number of synapses they can form with neurons and the blood-brain barrier [12]. Recently, there have been morphometric changes reported in astrocytes of suicidal humans [17]. The alternate hypothesis regards depression as a consequence of immune dysregulation [24].

Increasingly, astrocytes are being recognized as important modulators of immune function in the brains of individuals with aberrant behavior [25]. It is possible that elevated cortisol in individuals displaying aberrant behavior drives increased secretion of cytokines within brain [26]. Inflammation of brain has been shown to induce increased expression of quinolic acid [27] and toll-like receptors [28]. Indeed, it has been postulated that the upregulation of TLR genes could be the driving event in addictive behavior pathogenesis [29].

Changes in astrocyte morphology and function have been demonstrated in hormonal, neuropathological, and environmental conditions [17], [30]. Although many studies demonstrate astrocytic hypertrophy and gliosis in the setting of brain injury or neurodegeneration [31], there is evidence of decreased GFAP immunoreactivity and astrocytic atrophy in chronic disease [32].

To date, there has been little research to examine morphological changes and immune activation of astrocytes, especially in spontaneously occurring psychological disorders. The aim of this study is to examine these changes in astrocyte morphology and immune dysfunction in the setting of SIB in nonhuman primates. Our working hypothesis was that gray and white matter astrocytes would have altered morphology, combined with increased TLR expression.

## Materials and Methods

### Ethics Statement, Animal Housing and Selection of Tissues

Animals were maintained in Animal Biosafety Level 2 housing with a 12∶12- hour light:dark cycle, relative humidity 30% to 70%, and a temperature of 17.8 to 28.9°C. Water was available ad libitum, and a standard commercially formulated nonhuman primate diet (Lab Fiber Plus Monkey DT, 5K63, PMI Nutrition International, St. Louis, MO) was provided twice daily and supplemented daily with fresh fruit and/or forage material as part of the environmental enrichment program. All animals at TNPRC have environmental enrichment, widely used to improve welfare in captive macaques. Over the course of their life times, all subjects experienced some pair or group housing as well as periods of single housing. Each cage (Allentown, Inc., Allentown, NJ) measured 36 inches (91.4 centimeters) in height with 8.6 square feet (0.8 square meters) of floor space and contained a perch, a portable enrichment toy, a mirror, and a forage board for feeding enrichment. Practices in the housing and care of animals conformed to the regulations and standards of the PHS Policy on Humane Care and Use of Laboratory Animals, and the Guide for the Care and Use of Laboratory Animals. The Tulane National Primate Research Center (Animal Welfare Assurance # A4499-01) is fully accredited by the Association for the Assessment and Accreditation of Laboratory Animal Care-International. All animals are routinely cared for according to the guidelines prescribed by the NIH Guide to Laboratory Animal Care. The TNPRC conducts all research in accordance with the recommendations of the Weatherall report -‘‘The use of non-human primates in research’’. The Institutional Animal Care and Use Committee (IACUC) of the Tulane National Primate Research Center approved all animal-related protocols, including any treatments used with nonhuman primates. All animal procedures were overseen by veterinarians and their staff.

Each year, 15–20 animals (from a colony of approximately 5,000) are identified as exhibiting self-injurious behavior at TNPRC. These animals are “flagged” in the extensive records system, allowing them to be monitored prospectively and identified later for studies such as this one.

Animals were humanely euthanized by the veterinary staff at the TNPRC in accordance with endpoint policies. Euthanasia was conducted by anesthesia with ketamine hydrochloride (10 mg/kg) followed by an overdose with sodium pentobarbital. This method is consistent with the recommendation of the American Veterinary Medical Association guidelines.

For this retrospective study, tissues were selected solely on their availability in the TNPRC tissue archive. None of the animals in the study was receiving treatment for bacterial or parasitic infection. None of the macaques had been used for infectious or pharmacological studies. Frontal cortical tissue from 6 control, and 4 SIB rhesus macaques (Macaca mulatta) were used for this study, for a total of 10 animals (Table 1).

**Table 1:** List of SIB and Control Animals.

	Animal	Age(years)	Time of SIB(years)	Sex	Weight (kg)	Major Neuropathologic Diagnoses	Notes
**SIB**	CN59	19.52	1 day	Male	13.60	SIB	Aging study, obese
	GB61	5.65	Noted at necropsy	Male	6.40	SIB	
	NO61	18.31	3.59	Male	12.50	SIB	Euthanized due to dehydration
	EH70	3.00	1.51	Female	5.15	SIB	Euthanized for tissue collection, moderately atrophic thymus
**Control**	HP24	3.03	n/a	Male	3.42	n/a	
	EI93	5.31	n/a	Female	4.00	n/a	Diarrhea, dehydrated
	HT22	2.91	n/a	Male	5.55	n/a	
	HM63	3.04	n/a	Male	3.75	n/a	
	HN64	3.03	n/a	Male	4.85	n/a	
	HP24	3.03	n/a	Male	3.42	n/a	

### Immunohistochemistry

Formalin-fixed, paraffin-embedded tissues were sectioned at 6 µm and mounted onto positively charged glass slides. Sections were baked for overnight at 60°C, deparaffinized in xylene, and then rehydrated in graded concentrations of ethanol. Antigen retrieval was carried out for 20 min using a steamer and a citrate-based antigen unmasking solution (Vector Labs, Burlingame, CA). Tissues were blocked in blocking buffer (Dako) for one hour at room temperature before antibodies were applied. Tissues were incubated with TLR2 (ab24192, Abcam) and GFAP primary antibody (GA-5, Sigma) overnight at 4°C, washed three times with PBS with 0.2% bovine serum albumin (Santa Cruz) (PBS/BSA), and then incubated in the dark for 60 min at room temperature with secondary antibodies directly conjugated with Alexa 488 (green) or Alexa 568 (red) (Molecular Probes/Invitrogen, Carlsbad, CA). Sections were washed three times in PBS/BSA, cover-slipped with Prolong Gold with DAPI (Molecular Probes/Invitrogen), and imaged on a Nikon Eclipse TE2000-U microscope.

### Quantification of Astrocyte Morphology

**Figure 1:**
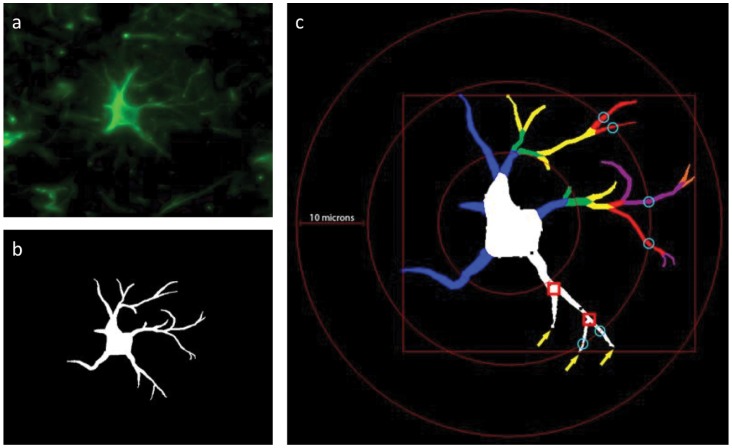
Schematic of Astrocyte Morphometrics Measured. Astrocytes, stained with GFAP were imaged using a fluorescent microscope (a). The image was then traced using the software (b) and the following morphological (c) parameters analyzed: cell body area (white), number of processes/dendrites, total arbor length (combined linear length), arbor volume (volume of 3D frusta), the number of nodes and the number of process tips.

All samples were coded and analyzed randomly by a researcher blinded to animal number and condition. Images of non-overlapping fields in frontal cortical sections were captured by fluorescence microscopy at 40X objective (Nikon Eclipse TE2000-U) and analyzed using Neurolucida software (MBF Bioscience). Using a nonhuman primate brain atlas, we identified areas consistent with the ACC, ventral to the cingulate sulcus and dorsal to the genu of the corpus callosum in paraffin-embedded frontal cortex sections. Protoplasmic grey matter astrocytes reside in layers 2–6 and are complex cells with numerous fine processes (Oberheim et al., 2012). We randomly selected astrocytes from layers 3–5 for our analysis. Fibrous white matter astrocytes identified along white matter tracts are generally less complex and were chosen randomly in the area of the anterior cingulate cortex. The fibrous white matter astrocytes were located significantly away from the grey-white matter interface so as not to be mistaken for Layer VI protoplasmic astrocytes. An average of 10 astrocytes with clear cell bodies and processes in both gray and white matter were chosen for reconstruction. The cells chosen were fully intact and did not have processes that touched the edges of the field. The resulting files generated by 2D reconstruction were analyzed with Neurolucida Explorer (MBF Bioscience), generating data of morphological measurements such as cell area, branching points (nodes), arbor length and volume (Figure 1a–b).

### Sholl Analyses

Sholl analysis was performed on the data by placing concentric rings 10 µm apart around the cell starting from the center of the cell body and radiating outward. Intersections were determined as points where the astrocytic processes crossed a concentric ring. Branching points (nodes) are expressed as a quantity per concentric ring area (Figure 1c).

### Quantification of TLR2 Expression

Images of double-labeled (GFAP and TLR2) sections in non-overlapping fields were captured by fluorescence microscopy (Nikon Eclipse TE2000-U). An average of five fields of astrocytes were imaged for both white and gray matter at 20X, and the number of GFAP and TLR2 double-labeled astrocytes was quantified and expressed as a percentage of the total number of GFAP-labeled cells.

### Statistical Analyses

Statistical analyses were performed using GraphPad Prism (version 5, GraphPad Software, La Jolla, CA). Normality was assessed by Kolmogorov-Smirnov test, and data that passed normality were analyzed by unpaired T-test. Data that were not distributed normally were assessed by Mann-Whitney test to determine significance between groups. Results are expressed as mean ± SEM. For all analyses, significance was set at p<0.05.

## Results

To begin a detailed assessment of astrocytic activation in self-injurious behavior, we have examined the anterior cingulate region of the frontal cortex in macaques identified with SIB. This was enabled using Neurolucida software and archived tissues. As outlined in Figure 1, GFAP expressing astrocytes were identified (a), and traced (b) using Neurolucida software. The software then determined numerous mathematical determinants of morphology, including cell body size, number of “dendrites”, bifurcations on dendrites, process tips, arbor length and volume.

### Cell Body Size

**Figure 2:**
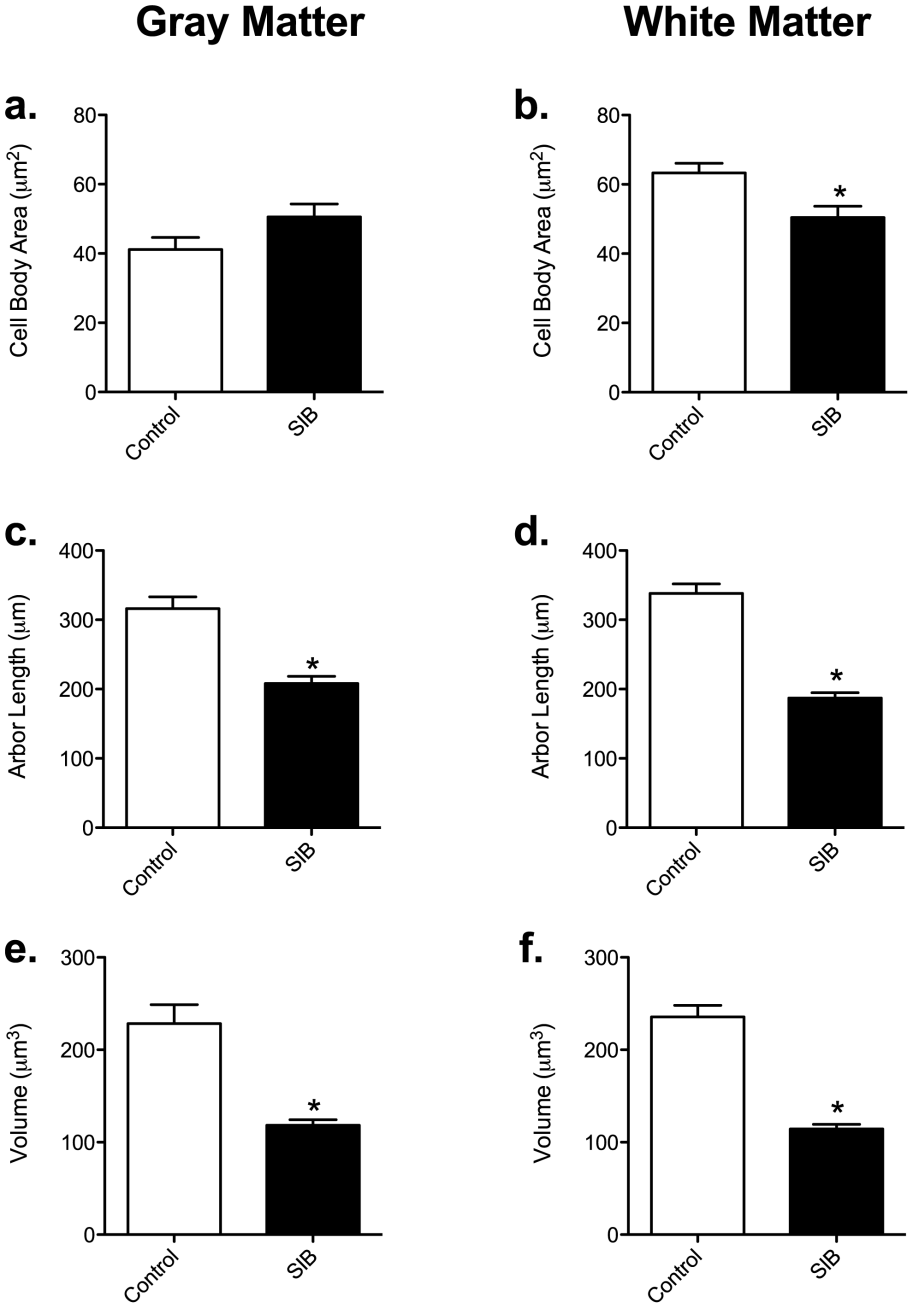
Astrocyte morphometrics in self-injurious behavior. The area of the astrocyte cell body was not significantly different in macaques with SIB in gray matter (a), but was decreased significantly in white matter astrocytes of SIB macaques (b). The cumulative length of the astrocyte processes was decreased in both gray (c) and white (d) matter, as was the overall volume of the processes (e & f, respectively). If a parameter is significantly different from the control, it is noted by an asterisk “*”.

No significant difference was found between gray matter astrocytes in control (41.17±3.493 µm^2^) and SIB (50.59±3.693 µm^2^) groups (Figure 2a). However, the size of the cell body of white matter fibrous astrocytes was significantly smaller in SIB animals (50.42±3.270 µm^2^) than control (63.32±2.743 µm^2^) (Figure 2b) indicating cytoplasmic atrophy.

### Astrocytic Processes

Segment length was measured from the edge of the cell body to the end of the process. The sum total of all of the processes radiating from the cell body was decreased significantly in the SIB groups (Figure 2c–d). This change was in both gray (SIB: 208.0±10.37 µm *vs* Control: 316.2±17.11 µm) and white (SIB: 186.9±7.753 µm *vs* Control: 338.3±13.39 µm) matter astrocytes. Additionally, the total volume of the astrocytic processes was decreased significantly in SIB gray (SIB: 118.3±6.121 µm *vs* Control: 228.4±20.47 µm; Figure 2e) and white matter astrocytes (SIB: 114.3±5.057 µm *vs* Control: 235.5±12.68 µm;Figure 2f).

### Astrocyte Complexity is Decreased in Frontal Cortex of SIB Macaques

**Figure 3:**
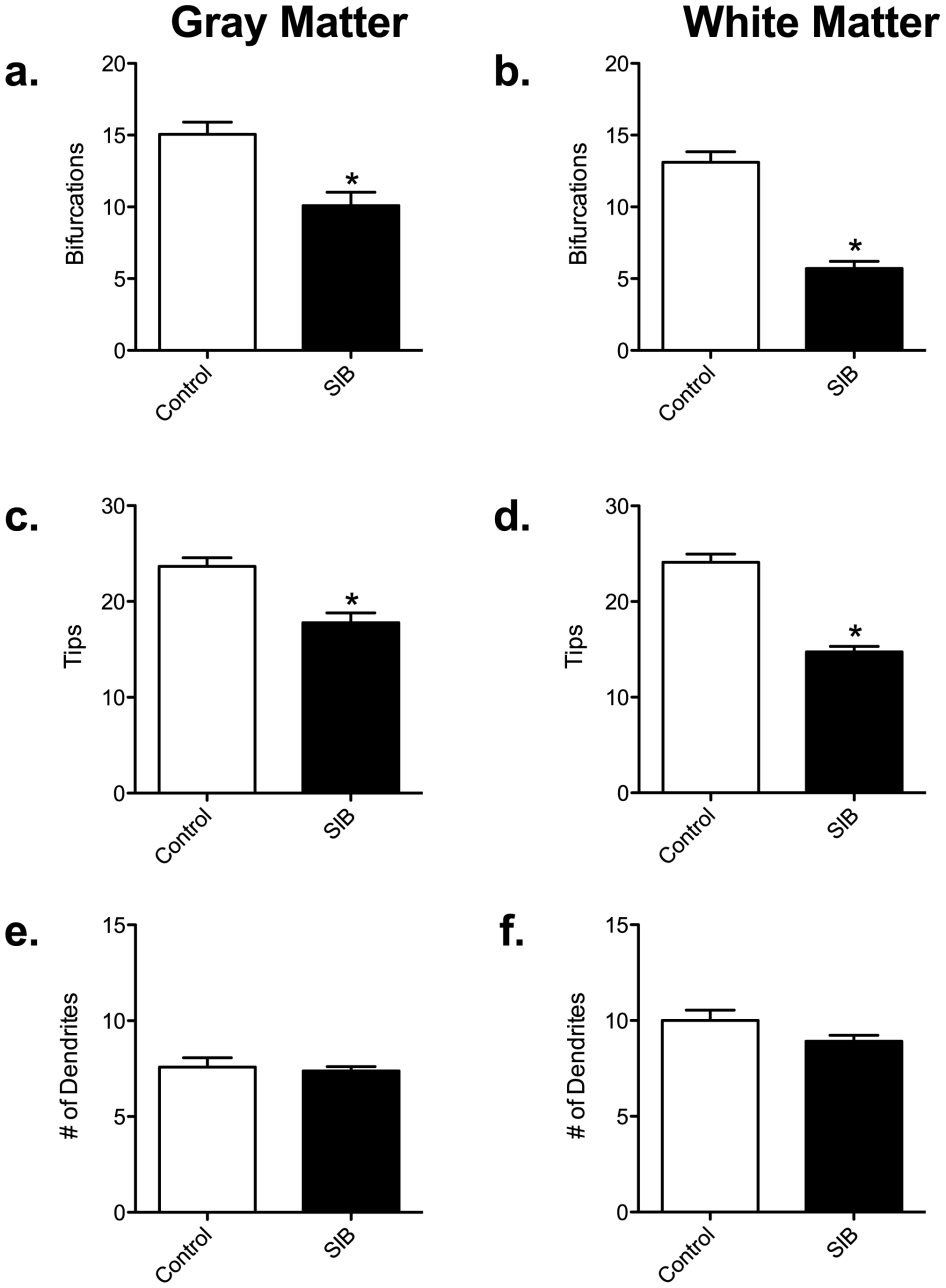
Astrocytes are less complex in self-injurious behavior. The number of bifurcations in the gray (a) and white (b) matter astrocytes was significantly decreased in the frontal cortex of macaques that had exhibited SIB. This correlated with a decreased number of tips, again in both gray (c) and white (d) matter. However, there was no alteration in the number of dendrites in either gray (e) or white (f) matter.

We assessed the complexity of astrocytes by calculating the number of nodes and process end points (Figure 3). The number of bifurcations (nodes) decreased significantly in gray (a, SIB: 10.09±0.9444 *vs* Control: 15.06±0.8370) and white (b, SIB: 5.714±0.4980 *vs* Control: 13.11±0.7296) matter astrocytes. Additionally, there was a reduction in the number of process end points (tip quantity) in gray (SIB: 17.79±1.030 *vs* Control: 23.66±0.8903) and white (SIB: 14.74±0.5744 *vs* Control: 24.11±0.8632) matter astrocytes compared with controls (Figure 3c–d, respectively). It was interesting that all of the above alterations in astrocyte morphology occurred in the absence of any change in the number of processes leaving the cell bodies in either gray or white matter astrocytes (Figure 3e–f).

### Sholl Analyses

**Figure 4:**
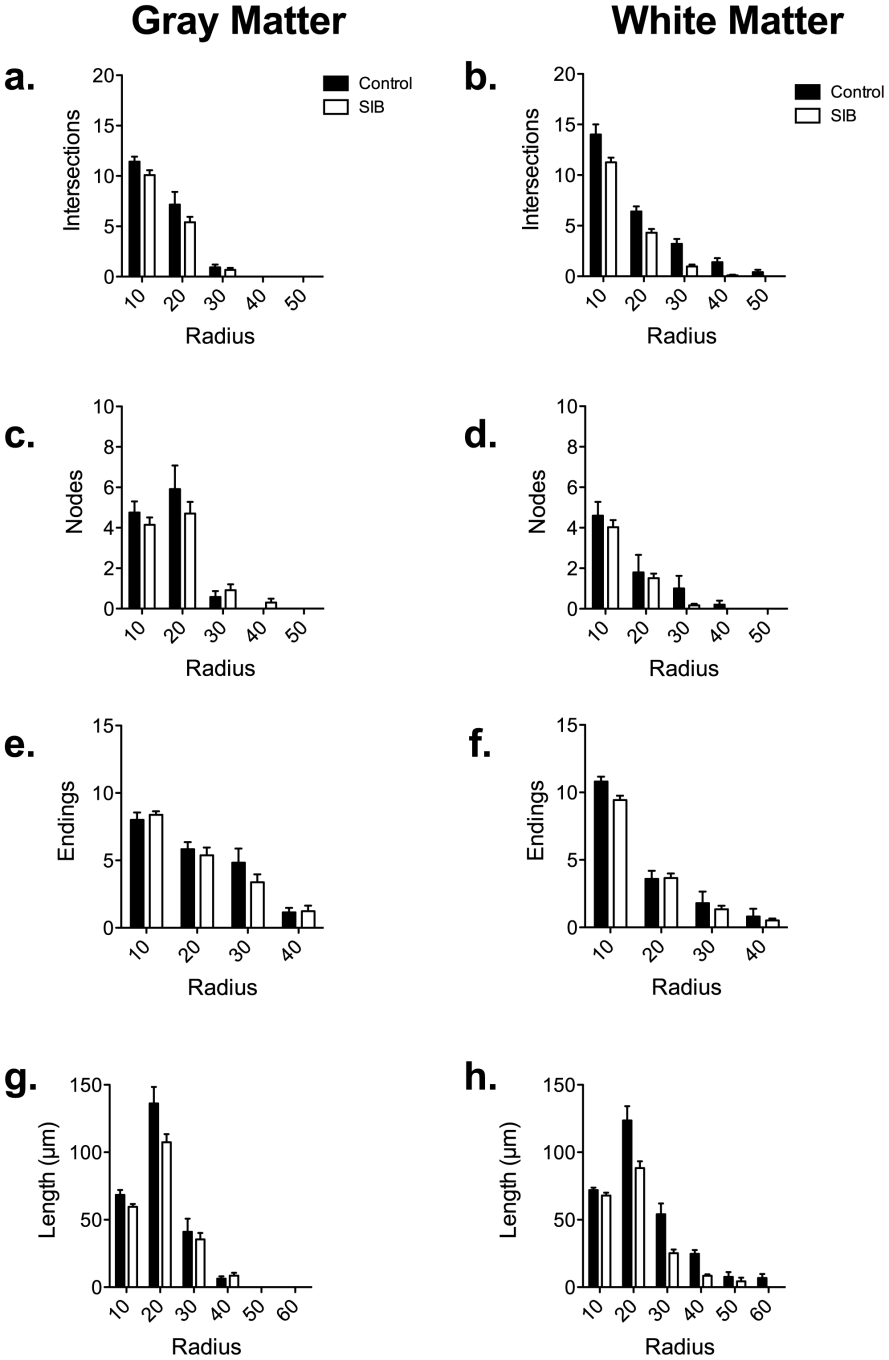
Sholl analysis of intersections, branching points, tips, and dendritic lengths made by white and gray matter astrocytes in SIB and control animals. The number of intersections, nodes or endings was not significantly decreased in astrocytes of macaques with SIB when examined by distance from the cell body (a–f). However, there was a significant decrease in the length of processes in white matter astrocytes (h).

Using Sholl analysis to further examine the complexity of the astrocytes, we quantified the length of astrocytic processes, as well as the number of intersections and nodes per 10 µm radius. While there was a general decrease in the number of intersections, nodes (bifurcations) and tips within the first 30 µm radii around the cell body of SIB gray and white matter astrocytes, these were not significant (Figure 4a–f). However, the total length of the cell arbor observed in white matter astrocytes was significantly decreased compared to controls (h, p = 0.0156).

### Astrocyte Atrophy is Associated with Immune Activation

**Figure 5:**
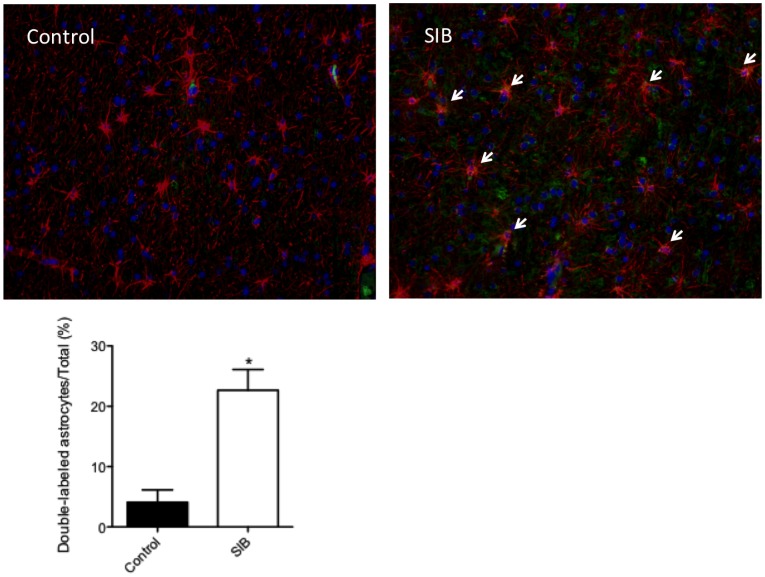
Immune activation of astrocytes in macaques with SIB. Control macaques have low levels of TLR2 expression in brain, including on astrocytes (a). In macaques with SIB (b), this level increases dramatically (from about 4% to 22%) indicating immune activation (c). TLR2 is represented in green, GFAP expression in red.

To determine if these morphologic alterations were associated with immune activation, double-labeled fluorescence microscopy was used to determine the expression of Toll-like receptor2 (TLR2) on astrocytes. Astrocytes in frontal cortex of control brains expressed very low levels of TLR2 (Figure 5a). However, TLR2 expression was increased on astrocytes of macaques identified with SIB (Figure 5b), by over fivefold (5c).

Overall, we observed significant decreases in process length (cell arbor) in gray and white matter astrocytes in primates with SIB. Gray and white matter astrocytes in SIB animals had decreased bifurcations compared to control animals. Sholl analysis showed a decrease in the complexity of SIB white matter astrocytes. Combined, these data suggest that in the setting of self-injurious behavior, astrocytes in the frontal cortex are both morphometrically and immunologically activated.

## Discussion

This study examined changes in astrocyte morphology and immune activation in rhesus macaques that exhibited self-injurious behavior. Unlike other animal models, depressive behavior, including SIB, occurs naturally in nonhuman primates and is not induced by experimental manipulation [33]. Symptomology can occur via chronic social subordination and chronic social isolation. The latter condition is associated with the spontaneous development of self-injurious behavior. SIB, which has been studied most thoroughly in the rhesus macaque, is observed in 5% to 13% of the caged population [34]. Severity of SIB in rhesus macaques ranges from biting that does not result in trauma to the skin to significant self-injury. Such behavior in macaques closely recapitulates self-harm in humans, presenting with reductions in heart rate and blood pressure following SIB bouts in both human and nonhuman primates [35]. In addition, this behavioral syndrome in primates can be ameliorated with a variety of medications commonly prescribed to combat aberrant behavior in humans [36], [37]. Macaques respond to clinically relevant drugs at doses and timescales similar to that observed in humans [11], making the nonhuman primate model ideal to determine the cellular and molecular mechanisms of depressive-like behaviors.

The nonhuman primate brain is the most similar to that of humans as regards to architecture and patterns of connectivity, and has recently been recognized as the closest model to human depression [33]. Intriguingly, primates also have unique astrocyte subtypes not present in other taxa [13]. Thus, the nonhuman primate model offers a novel translational system to examine the cellular and molecular aspects of aberrant behavior, and are the only ethically sound model for self-harm/suicide. While the macaque has been used to model self-harm in humans [10], it has not, to the authors’ knowledge, been examined at the level of astrocyte activation.

Although the potential importance of glial cell activation in mental health has been appreciated [13], [17], [38], no approach to date has been effective in moving the research beyond the status quo, which is little or no appreciation of the combined activation of innate immune function of astrocytes with how they interact with other cell types. For example, Crews has postulated innate immune activation [29], and Torres-Platas has recently described altered morphometrics in one reward center in depressed humans [17]. However, these studies have approached each component of astrocyte activation in isolation.

Here, we provide detailed neuroanatomical analysis of fibrous white and protoplasmic gray matter astrocytes in the frontal cortex. We found that in the setting of SIB, cortical gray and white matter astrocytes in the frontal cortex of rhesus macaques show marked atrophy in process length, number, volume, and complexity. Increased cell body area, number of nodes and increased process length in white matter have recently been observed in astrocytes of depressed suicides [17]. We were therefore intrigued to discover that astrocytes in macaques with SIB were noted to have reduced numbers of tips, nodes, arbor length and volume in both grey and white matter indicating subtle changes in morphometrics. That these changes in GFAP expression were observed in both white and gray matter is perhaps unusual, as it has been speculated that oligodendrocyte pathologies account for white matter abnormalities observed previously [39]. Therefore, these changes in astrocytes in both gray and white matter are novel. It is not certain whether these changes in astrocyte morphology are an early pathological event or a consequence of profound immune or hormonal activation.

It has been demonstrated that astrocytes display dynamic plasticity in their distal processes in response to changes in their extracellular environment [40]. We have recently shown that, in the setting of SIV infection (without encephalitis) and SIV-induced encephalitis, gray and white matter astrocytes retract their processes resulting in an overall decreased total arbor (Lee et al, under review). Curiously, in SIB macaques, there was no such decrease in the number of processes, although the arbor was significantly decreased (Figure 2). However, the decreased number of tips would decrease the amount of processes available for endothelial and neuronal connection. Regardless of the site of retraction, these structural changes would lessen the number of foot processes contacting endothelial cells and neurons, creating BBB dysfunction and altered neuronal homeostasis. A major consequence of astrocyte dysfunction would then result in a loss of neuronal support and protection, including disruption of glutamate homeostasis, which leads to synapse dysfunction and neurotoxicity [38], [41], [42].

Additionally, astrocytes react to various CNS insults through cell body and process hypertrophy, upregulation of intermediate filament expression, and glial scar formation [43]. However, glial scarring is rarely observed in the absence of infectious agents, and the data on astrocyte cell body size are conflicting. Decreased cell body size was observed in the hippocampus of chronic stressors [12], but increased in the anterior cingulate cortex of depressed suicides [17]. The atrophy observed in SIB macaques provide further evidence that SIB is distinct from depression/suicide [44].

We have recently noted that astrocytes in frontal cortex of rhesus macaques have altered morphologic parameters following infection with SIV, regardless of whether there is still virus present in the brain (Lee et al, under review). This was combined with increased TLR2 expression. TLRs are also dysregulated in brain of addicted individuals [29], including those who abuse psychostimulants [45]. Thus, it was logical that there could be an increase in expression of TLR2 on astrocytes in SIB macaques.

In the current study, we examined astrocytes from frontal cortices in SIB-affected macaques to obtain a general understanding of changes occurring in the brain in response to self-harm. Gray and white matter astrocytes in the setting of SIB showed decreases in complexity compared to control astrocytes. Curiously, neither gray nor white matter astrocytes showed an increase in cell body size: indicating no hypertrophy. Further analyses examining changes in specific cortical areas are warranted.
